# FBX8 Acts as an Invasion and Metastasis Suppressor and Correlates with Poor Survival in Hepatocellular Carcinoma

**DOI:** 10.1371/journal.pone.0065495

**Published:** 2013-06-27

**Authors:** Feifei Wang, Yudan Qiao, Jiang Yu, Xiaoli Ren, Jianmei Wang, Yi Ding, Xiaojing Zhang, Wenhui Ma, Yanqing Ding, Li Liang

**Affiliations:** 1 Department of Pathology, Nanfang Hospital, Southern Medical University, Guangzhou City, Guangdong Province, People's Republic of China; 2 Department of Pathology, School of Basic Medical Sciences, Southern Medical University, Guangzhou City, Guangdong Province, People's Republic of China; 3 Department of General Surgery, Nanfang Hospital, Southern Medical University, Guangzhou, Guangdong Province, People's Republic of China; 4 Department of Pathology, Shenzhen University, Shenzhen City, Guangdong Province, People's Republic of China; 5 Department of Radiotherapy, Nanfang Hospital, Southern Medical University, Guangzhou City, Guangdong Province, People's Republic of China; University of Hong Kong, Hong Kong

## Abstract

**Background:**

F-box only protein 8 (FBX8), a novel component of F-box proteins, is lost in several cancers and has been associated with invasiveness of cancer cells. However, its expression pattern and role in the progression of hepatocellular carcinoma remain unclear. This study investigated the prognostic significance of FBX8 in hepatocellular carcinoma samples and analyzed FBX8 function in hepatocellular carcinoma cells by gene manipulation.

**Methodology:**

The expression of FBX8 was detected in 120 cases of clinical paraffin-embedded hepatocellular carcinoma tissues, 20 matched pairs of fresh tissues and five hepatocellular carcinoma cell lines by immunohistochemistry with clinicopathological analyses, real-time RT-PCR or Western blot. The correlation of FBX8 expression with cell proliferation and invasion in five HCC cell lines was analyzed. Moreover, loss of function and gain of function assays were performed to evaluate the effect of FBX8 on cell proliferation, motility, invasion *in vitro* and metastasis *in vivo*.

**Conclusions:**

We found that FBX8 was obviously down-regulated in HCC tissues and cell lines (P<0.05). The FBX8 down-regulation correlated significantly with poor prognosis, and FBX8 status was identified as an independent significant prognostic factor. Over-expression of FBX8 decreased proliferation, migration and invasion in HepG2 and 97H cells, while knock-down of FBX8 in 7721 cells showed the opposite effect. FBX8 negatively correlated with cell proliferation and invasion in 7701, M3, HepG2 and 97H cell lines. In vivo functional assays showed FBX8 suppressed tumor growth and pulmonary metastatic potential in mice. Our results indicate that down-regulation of FBX8 significantly correlates with invasion, metastasis and poor survival in hepatocellular carcinoma patients. It may be a useful biomarker for therapeutic strategy and control in hepatocellular carcinoma treatment.

## Introduction

Hepatocellular carcinoma (HCC) is a common malignancy worldwide, but especially in China and other East Asian countries [1], [2]. Although survival of patients with HCC has improved due to advances in surgical techniques and perioperative management, long-term survival after surgical resection remains low due to the high rate of recurrence and metastasis [3]–[4]. Although some clinicopathological features of HCC, such as tumor multifocality, vascular invasion and tumor size, are useful to evaluate the prognosis of HCC patients, they can't meet clinical requirements for precise prediction of HCC course [4]–[5]. Therefore, it is of great interest to search for valuable factors for prognosis prediction and novel therapeutic strategies.

The ubiquitin-dependent proteolytic pathway is an important mechanism of protein abundance regulation in eukaryotes. F-box proteins are critical components of the SCF uniquitin-protein ligase complex and are involved in the ubiquitin-dependent proteolytic pathway. So far, more than 70 putative F-box proteins have been identidied in human genome, although the function and their substrates of most F-box proteins remain elusive [6]. Only several members of F-box proteins such as Skp2 and Fbw7 have been well-studied in cancer. Recent studies revealed that Skp2 and Fbw7 were closely associated with tumor progression and metastasis [7]–[9]. FBX8 (F-box only protein 8, also named FBXO8) is a novel component of F-box proteins, which contains an F-box domain and a putative Sec7 domain. FBX8 was originally identified as a Skp1-binding protein [10], [11]. It has E3 ligase activity mediating the ubiquitination of the GTP-binding protein ARF6 [12]. Moreover, FBX8 over-expression could inhibit ARF6-mediated cell invasion activity in breast cancer cells [12]. FBX8 was found to be a novel c-Myc binding protein and c-Myc induced cell invasive activity through the inhibition of FBX8 effects on ARF6 function [13]. Expression of FBX8 has been reported to be lost in some tumor cells, such as breast cancer and lung cancer cells [12], [14].

Till now, the molecular and biological functions of FBX8 in the development and progression of HCC remain unknown. To address this question, we evaluated FBX8 expression in HCC cell lines and clinical tissues, investigated the effect of FBX8 on the proliferation, invasion and metastasis of HCC cells.

## Materials and Methods

### Immunohistochemical staining

The sections were deparaffinized and rehydrated, and endogenous peroxidase was inhibited with 0.3% H_2_O_2_ methanol. For antigen retrieval, slides were boiled in 0.01M, pH 6.0 sodium citrate buffer for 15 min in a microwave oven. After blocked with the 5% normal goat serum, primary anti-FBX8 monoclonal antibody (Abcam, 1∶200) in blocking buffer (1∶50) was applied and the slides were incubated at 4°C overnight. After incubation with secondary antibody, the visualization signal was developed with DAB. The stained tissue sections were reviewed and scored separately by two pathologists blinded to the clinical parameters.

### Construction of plasmids and transfection

For overexpression of FBX8, FBX8 lentivirus expressing vector (GeneCopier, USA) was transfected into lentiviral packaging cell lines 293T cells. Then 1 mL of viral supernatant containing 4Ag of polybrene was added into HCC cell lines for stable transduction. After 14 days, puromycin-resistant cell pools were established. After 72 h, Western blot was performed to detect FBX8 expression. For depletion of FBX8, human shRNA1 and shRNA2 sequences (sense, 5′-CGA AAG AAC AGG AAG GAUU dTdT-3′; sense, 5′-CCA AAU GCA CUG AGA GAAU dTdT-3′) were cloned into Pgenesil-1 plasmid (Genesil Biotechnology, Co Ltd). A scramble siRNA (5′-AAT CGC ATA GCG TAT GCC GTT-3′), which has no homology with the mammalian mRNA sequences, was inserted into Pgenesil vector as control. Cells were all transfected with 3 µg of plasmids using Lipofectamine2000 according to the instructions (Invitrogen, USA). Western blot was performed to detect FBX8 expression.

### Cell proliferation assay

The cells were seeded in 96-well plates (1×10^4^ cells/ml) with 100 µl cell suspension in each well and incubated for 7 days respectively. MTT assay was performed by adding 20 µl of MTT (5 mg/ml; Promega, Madison city, USA) for 4 hours until a purple precipitate was visible. Precipitates were dissolved in 150 µl of DMSO. The absorbance value of each well was measured with a microplate reader set at 570 nm. Each experiment was repeated three times.

### In vitro invasion assay

Invasion Boyden Chamber is inserted with 8 µm-pores in the Polyethylene terephthalate membrane which has been coated by matrigel (BD Biosciences, Foster city, USA). RPMI 1640 with 100 ml/L fetal bovine serum was added to the lower compartment as the chemotactic factor. Then 1.5×10^5^ tumor cells in serum-free RPMI 1640 were added to the upper compartment of the chamber. Each cell group was plated in 3 duplicate wells. After incubation for 48 hr, cells that had migrated through the membrane and stuck to the lower surface of the membrane were fixed and stained with hematoxylin. Finally, the cells in lower compartment of the chamber that had invaded the lower sides of the membrane were counted under a light microscope in 5 random visual fields (200×).

### Metastasis in the mouse model

To evaluate in vivo tumor growth, 1×10^7^ cells were injected subcutaneously into the left flank or right flank of nude mice (n = 5 per group). Tumors were measured with calipers to estimate volume from day 5 to day 28 after injection. To evaluate in vivo metastasis, 5×10^5^ cells were injected into the tail veins of 6- to 9-week-old NOD/SCID mice (n = 6 per group). After 8 weeks, the animals were euthanized, and various organs from the thoracic, peritoneal and retroperitoneal cavities were removed, rinsed, fixed and subjected to pathological examination. The number of tumor colonies was determined by using a dissecting microscope.

Materials of HCC cell lines and clinical tissue specimens, and methods of evaluation of immunohistochemical staining, real-time RT-PCR, Western blotting, plate colony formation array and in vitro motility array were seen in Method S1.

## Results

### Immunohistochemical analysis of FBX8 in human HCC tissues

**Figure 1:**
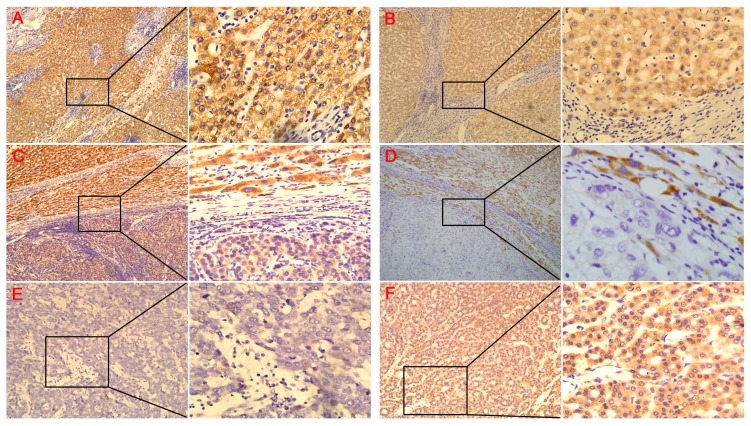
FBX8 is down-regulated in clinical paraffin-embedded HCC tissues. (A) Strong positive expression of FBX8 in adjacent normal livers (×100, ×400). (B) Positive expression of FBX8 in the cirrhotic livers (×100, ×400). (C) Week expression of FBX8 in HCC tissues while strong positive expression in adjacent livers (×200, ×400). (D) Negative expression of FBX8 in HCC tissues compared with strong positive expression in adjacent livers (×200, ×400). (E) Negative expression of FBX8 in HCC tissues with low differentiation (×200, ×400). (F) Weak expression of FBX8 in HCC tissues with high differentiation (×200, ×400).

To assess the expression of FBX8 in clinical paraffin-embedded HCC tissues, IHC was performed in 106 cases of HCC tissues. The subcellular location of FBXO8 was observed in the cytoplasm of hepatocytes (Figure 1A–D). The staining signal of FBX8 was observed mainly in adjacent normal livers or cirrhotic livers (Figure 1A, 1B), and no signals or only weak signals were detected in HCC tissues (Figure 1E, 1F). Among 106 samples, 83 cases of them (78.3%) exhibited low-expression of FBX8 (0 to 1+) and high-expression of FBX8 (2+ to 3+) was found in the other 23 cases (21.7%, Table 1). The expressions of FBX8 were significantly lower in HCC tissues than in adjacent normal livers or cirrhotic livers respectively (Z = −7.038, P<0.001; Z = −5.047, P<0.001). There was no significant difference of FBX8 expression between HCC tissues with cirrhosis and those without cirrhosis (λ = 1.986, P = 0.575, Table S1 in File S1). The relationship between clinicopathologic features and FBX8 expression in HCC was summarized in Table 1. FBX8 expression was correlated strongly with differentiation (P = 0.008) and serum AFP (P = 0.005). The above results indicate that the down-regulation of FBX8 may be associated with the progression of HCC.

**Table 1:** Relationship between FBX8 expression and clinicopathologic features of HCC patients.

Features	Total Number	High expression	Low expression	P	λ^2^
All case	106	23	83		
Age				0.331	0.945
<55		14	54		
> = 55		11	27		
Gender				0.637	0.223
Male		21	71		
Female		4	10		
Tumor size				0.924	0.009
<5 cm		12	38		
> = 5 cm		13	43		
Differentiation				***0.008***	9.593
Well		11	16		
Moderate		11	53		
Poor		3	16		
Cirrhosis				0.689	0.161
N		13	43		
Y		12	38		
Distant Metastasis				0.068	3.325
N		17	52		
Y		8	29		
Relapse				0.673	0.178
N		13	46		
Y		12	35		
Portal Vein Thrombosis				0.885	0.021
Y		4	12		
N		21	69		
Dissemination				0.321	1.024
N		14	36		
Y		11	45		
HBsAg status				0.09	2.873
+		2	19		
−		23	62		
Serum AFP				***0.005***	8.062
>25 ng		8	29		
≦25 ng		17	52		

### Correlation between FBX8 expression and patients' survival

**Figure 2:**
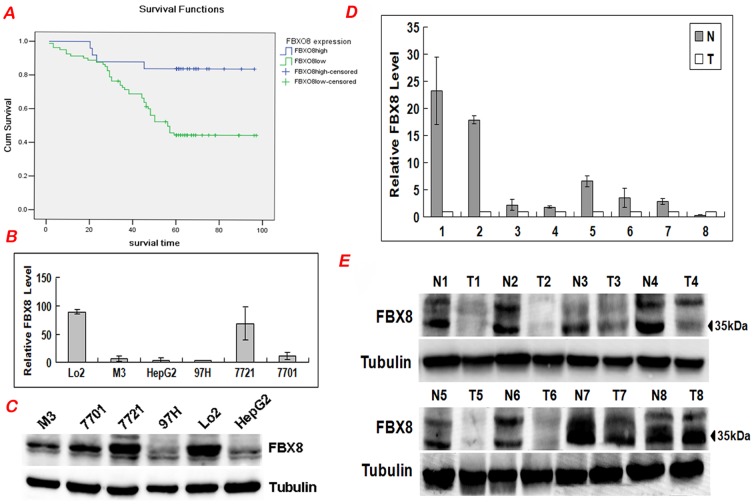
FBX8 is down-regulated in HCC cell lines and fresh HCC tissues. (A) Kaplan-Meier survival analysis of primary HCC patients with high and low FBX8 expressions. (B) Real-time RT-PCR analysis of FBX8 in six cell lines. The relative mRNA levels with the use of control LO2 were normalized to 1. (C) Western blotting analysis of FBX8 in six cell lines. (D) Real-time PCR analysis of FBX8 expression in 8 paired HCC tissues. (E) Western blotting analyses of FBX8 expression in the same 8 paired HCC tissues. N = normal mucosa and T = tumor.

The prognostic effect of FBX8 on HCC patients' overall survival was compared between patients with high and low FBX8 protein levels. By Kaplan-Meier curve assessment, patients with low FBX8 protein level had a significantly lower 5-year survival rate than those with high FBX8 protein level (P = 0.002, Figure 2A). From univariate analysis, the significant prognostic factors were FBX8 expression (P = 0.005), portal vein thrombosis (P<0.001), differentiation (P = 0.001), distant metastasis (P = 0.004), dissemination (P<0.001, Table S3 in File S1). Multivariate analysis results showed that FBX8 expression, portal vein thrombosis, differentiation and dissemination might play a role in predicting the overall survival in HCC patients (P<0.05, Table S3 in File S1). These results show that FBX8 expression is an independent prognostic marker for survival of HCC patients.

### FBX8 expressions in HCC cell lines and fresh HCC tissues

To further validate the down-regulation of FBX8 in HCC, real-time RT-PCR and Western blot were performed to detect the expression level of FBX8 in one hepatocyte cell line, five HCC cell lines and 16 matched pairs of fresh CRC tissues. Results showed that all the five HCC cell lines showed lower expression of FBX8 in comparison with hepatocyte cell line. Among five HCC cell lines, FBX8 expression was highest in 7721 cells, and gradually decreased in 7701, M3, HepG2 and 97H cells (Figure 2B). In addition, the level of FBX8 protein expression in each cell by Western blot coincided precisely with that of the mRNA level (Figure 2C).

FBX8 was obviously down-regulated in 16 cases of HCC tissues compared to adjacent noncancerous livers by real-time RT-PCR (T = 2.541, P = 0.017). Only two HCC tissues exhibited the almost identical or down-regulated expression of FBX8 compared to adjacent noncancerous livers (Figure 2D). Western blot results both revealed the similar changes in down-regulation of FBX8 protein in HCC tissues (Figure 2E). These results also confirm the decreased expression of FBX8 in HCC.

### Effect of FBX8 on cell proliferation, migration and invasion in vitro

**Figure 3:**
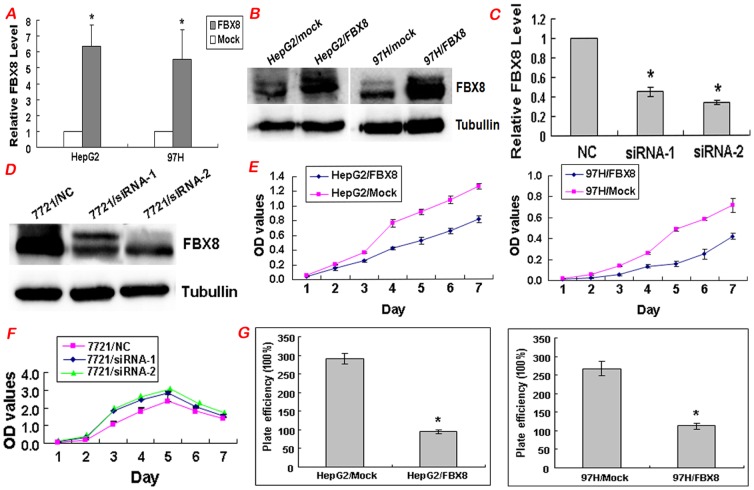
FBX8 inhibits proliferation of HCC cells in vitro. (A) FBX8 expression in FBX8 overexpressing 97H and HepG2 cells by real-time RT-PCR. (B) FBX8 expression in FBX8 overexpressing 97H and HepG2 cells by Western blotting. (C) FBX8 expression in FBX8 depleting 7721 cells by real-time RT-PCR. (D) FBX8 expression in FBX8 depleting 7721 cells by Western blotting. (E) Effect of ectopic FBX8 on cell proliferation in vitro by MTT assay. (F) Effect of FBX8 knockdown on cell proliferation in vitro by MTT assay. (G) Effect of ectopic FBX8 on cell proliferation in vitro by colony formation assay. *P<0.05.

**Figure 4:**
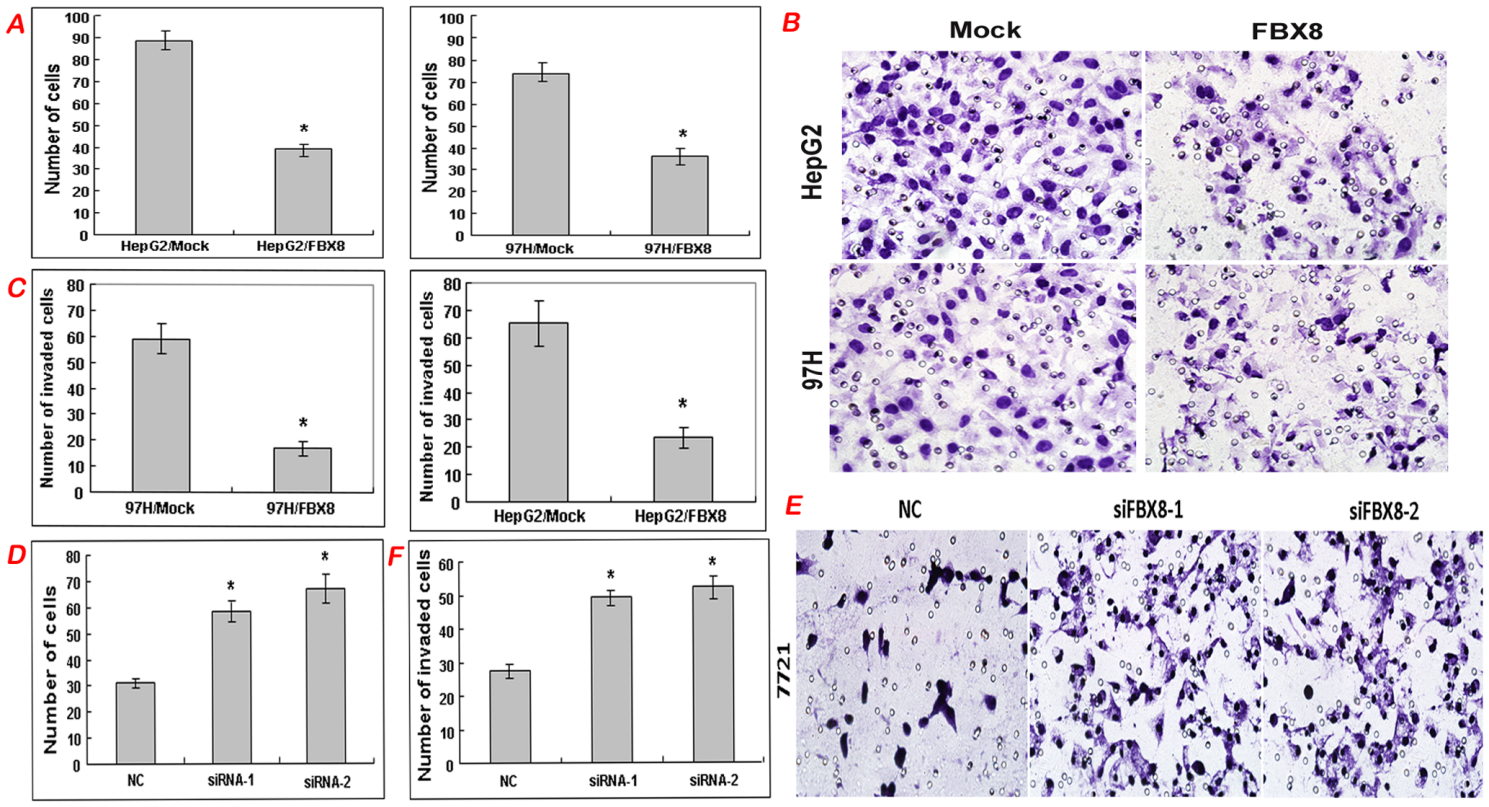
FBX8 suppresses motility and invasion of HCC cells in vitro. (A) Effect of ectopic FBX8 on cell motility in vitro. (B, C) Effect of ectopic FBX8 on cell invasion in vitro. Morphological comparison of cells penetrating the artificial basement membrane was also shown. (D) Effect of FBX8 knockdown on cell motility in vitro. (E, F) Effect of FBX8 knockdown on cell invasion in vitro. Morphological comparison of cells penetrating the artificial basement membrane was also shown. *P<0.05.

To determine the role of FBX8 in the progression of HCC, we performed gain of function and loss of function to investigate changes in malignant potentials of HCC cells in vitro. According to endogenous FBX8 expression in five HCC cells, we chose HepG2 and 97H cell lines for over-expression of FBX8 lentiviral vector, and 7721 cell line for FBX8 knockdown by RNA interference. Western blot and real-time RT-PCR validated high transfection efficiency (Figure 3A–3D). MTT assay showed that forced expression of FBX8 caused a significant decrease of the proliferation rate in HepG2 and 97H cells (P<0.05, Figure 3E), while FBX8 depletion increased the proliferative abilities of 7721 cells (P<0.05, Figure 3F). The inhibitory effect of FBX8 on proliferative abilities of HCC cells was further validated by the colony formation assay (P<0.05, Figure 3G). Forced expression of FBX8 markedly blocked the motility abilities of HepG2 and 97H cells (P<0.05, Figure 4A). Results of Boyden Chamber assay showed that the cells penetrating the artificial basement membrane in FBX8-overexpressing cells were less than those in mock cells (P<0.05, Figure 4B–C). On the contrary, FBX8-depleting cells displayed a marked increase of motility ability (P<0.05, Figure 4D) and invasive ability (P<0.05, Figure 4E–F). Next, we detected the proliferation and invasion *in vitro* in five HCC cells and found that cell proliferation and invasion were progressively increased in 7701, M3, HepG2 and 97H cells, whereas endogeneous FBX8 expression showed the opposite tendency in those cells. 7721 cells had the highest FBX8 expression, proliferation but relatively low invasive ability (Figure S1A–B). Thus we validated that FBX8 negatively correlated with cell proliferation and invasion in four HCC cell lines. The above results suggest that FBX8 negatively regulates proliferation, motility and invasion *in vitro* of HCC cells.

### Effect of FBX8 on tumor growth and metastasis in vivo

**Figure 5:**
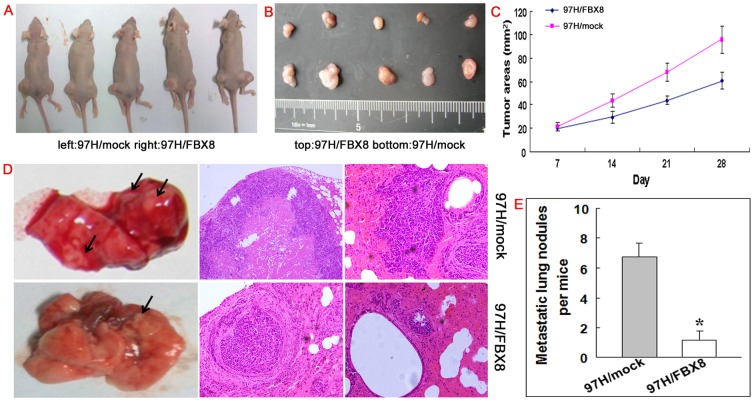
FBX8 inhibits tumor growth and metastasis of HCC cells in vivo. (A, B) Subcutaneous tumors of mice injected with 97H/mock and 97H/FBX8 cells. (C) Effects of FBX8 on subcutaneous tumor growth by MTT assay. (D) Pulmonary metastases of mice injected with 97H/mock and 97H/FBX8 cells. (E) Number of metastatic intestinal or hepatic nodules per mice. The number of metastatic nodules in individual mice was counted under the microscope. * P<0.05.

To assess the effect of FBX8 on tumor growth *in vivo*, we implanted FBX8-expressing 97H cells and mock cells subcutaneously into nude mice respectively, and then monitored the growth of the resultant primary tumors. Palpable tumors were first detected in all mice by day 7 after injection. At day 28, tumors in mice injected with 97H/FBX8 cells were smaller than mock cells (P<0.05, Figure 5A–C). To test the effect of FBX8 on metastasis *in vivo*, we adopted the experimental mouse model injecting FBX8-expressing 97H cells and mock cells into the tail veins. We observed that 67% of mice injected with mock cells (n = 4 of 6 cases) developed lung metastasis. However, in the 97H/FBX8 group, only 17% (n = 1 of 6 cases) of mice had lung metastasis. No other detectable tumor metastasis was found in 97H/FBX8 and mock groups. Many large pulmonary metastatic nodules with central necrosis were observed in mice injected with mock cells, while a few small nodules were detected in 97H/FBX8 group (Fig. 5D). The number of metastatic lesions in 97H/FBX8 group was significantly less than mock group (P<0.05, Fig. 5E). These results, collectively, indicate that FBX8 expression profoundly decreases tumor growth and metastasis *in vivo*.

## Discussion

FBX8 is a novel member of the F-box protein family which is characterized by an approximately 40 amino acid motif, the F-box. The F-box proteins constitute one of the four subunits of the ubiquitin protein ligase complex called SKP1-cullin-F-box (SCFs), which is involved in phosphorylation-dependent ubiquitination [15]. Till now, only three recent papers have discussed the function of FBX8 in cancer [12]–[14]. FBX8 mediates uniquitination of ADP-ribosylation factor 6 (ARF6) and exhibits ARF-GEF (guanine nucleotide exchange factor) activity [12]. FBX8 expression is impaired in breast cancer and lung cancer cells [12], [14]. Moreover, FBX8 inhibits ARF6-mediated cell invasion activity in breast cancer [12] and c-Myc stimulates cell invasion by inhibiting FBX8 function [13]. The expression pattern and role of FBX8 in the progression of HCC have not been illustrated.

In this study, we first detected the expression of FBX8 in 106 cases of clinical paraffin-embedded HCC tissues, 5 HCC cell lines and 16 matched clinical fresh HCC tissues. IHC results showed that the positive signaling of FBXO8 was observed in the cytoplasm of hepatocytes, which was consistent with the study from Cho et al [13]. They also raised that FBX8 protein colocalized with c-Myc in the nucleus after cotransfection with the wide-type c-Myc expression plasmid [13]. FBX8 expression was significantly lower in HCC tissues than adjacent normal livers or cirrhotic livers (P<0.001). We also found that FBX8 was down-regulated in four HCC cell lines as well as in 16 matched clinical fresh tissues (P<0.05). Many F-box proteins such as FBXW7, FBXL2, FBX4 and FBXO11 are found to be lost in a wide range of human cancers, and generally considered as tumor suppressor genes [16]–[20]. Our data also clearly demonstrate the decreased expression of FBX8 in HCC.

Then we examined the clinicopathological values of FBX8 in HCC tissues. In many clinicopathologic features, differentiation and serum AFP were correlated strongly with FBX8 expression (P<0.01). Low FBX8 protein level was a significant prognostic factor for poor overall survival in HCC patients (P<0.001). Moreover, FBX8 expression, portal vein thrombosis, differentiation and dissemination were singled out as four marked and independent prognostic factors relative to overall survival from multivariate analysis (P<0.05). Besides FBX8 expression, the three other factors are well-acknowledged indicators in the progression of HCC [21]–[23]. These data indicate that FBX8 expression is associated with the progression of HCC and an independent prognostic marker for survival of HCC patients.

Recent evidence suggests an important role for several F-box proteins in modulating cancer progression. FBXW7 can ubiquitinate c-Myc and cyclin E, resulting in exit from the cell cycle [24] and subsequently inhibit cell proliferation of colorectal cancer [25]. Human F-box protein hCdc4 also targets cyclin E for proteolysis [26]. Moreover, inactivation of Fbxw7 attenuates TGF β-dependent regulation of cell growth and migration of breast cancer [27]. miR-223 targets FBXW7/hCdc4 expression at the post-transcriptional level and appears to regulate cellular apoptosis, proliferation, and invasion in gastric cancer [28]. S-phage kinase-associated protein 2 (Skp2) and Myc coordinate to induced RhoA transcription and promote breast cancer metastasis [29], [30]. We next explored the possible function of FBX8 in the progression of HCC. The results showed that forced expression of FBX8 in HepG2 and 97H cells increased cancer cell proliferation, motility and invasion in vitro. Depletion of FBX8 in 7721 cells showed the opposite effects. We also showed that FBX8 negatively correlated with cell proliferation and invasion in 7701, M3, HepG2 and 97H cell lines, but not in 7721 cells, which had the highest FBX8 expression, proliferation and relatively low invasive ability. In vivo functional results showed that ectopic FBX8 obviously suppressed subcutaneous tumor growth and yielded more and larger metastatic nodules in the lung compared to mock cells. Thus, the above results provide evidence that FBX8 can function as a suppressor in invasion and metastasis of HCC. The molecular mechanisms of FBX8 in HCC progression need to be further investigated.

In summary, our study demonstrates that down-regulation of FBX8 in HCC correlates with poor survival of patients. All of the functional experiments confirm FBX8 as a tumor suppressor in the progression of HCC. The preferential down-regulation of FBX8 in HCC patients with invasion and metastasis suggested that FBX8 may be a significant biomarker for HCC progression. Inhibitors targeting FBX8 would give considerable therapeutic potential in the treatment of HCC.

## Supporting Information

Figure S1 **The proliferative and invasive abilities of five HCC cells in vitro.** (A) Cell proliferation of five HCC cells was detected by MTT assay. (B) Cell invasion of five HCC cells was examined by Boyden invasion chamber.
(TIF)

File S1 Table S1. Expression of FBX8 in HCC tissues, cirrhotic liver and adjacent normal liver tissues. Table S2. Clinical characteristics of 106 cases of HCC patients. Table S3. Univariate and multivariate analyses of individual parameters for correlations with overall Survival rate: Cox proportional hazards model.
(DOCX)

Methods S1 **Supporting material.**
(DOC)
