# Supplementary figures and images for: FBX8 Acts as an Invasion and Metastasis Suppressor and Correlates with Poor Survival in Hepatocellular Carcinoma
Source: PLoS One. 2013 Jun 27;8(6):e65495. doi: 10.1371/journal.pone.0065495 (PMC3694991; doi:10.1371/journal.pone.0065495)

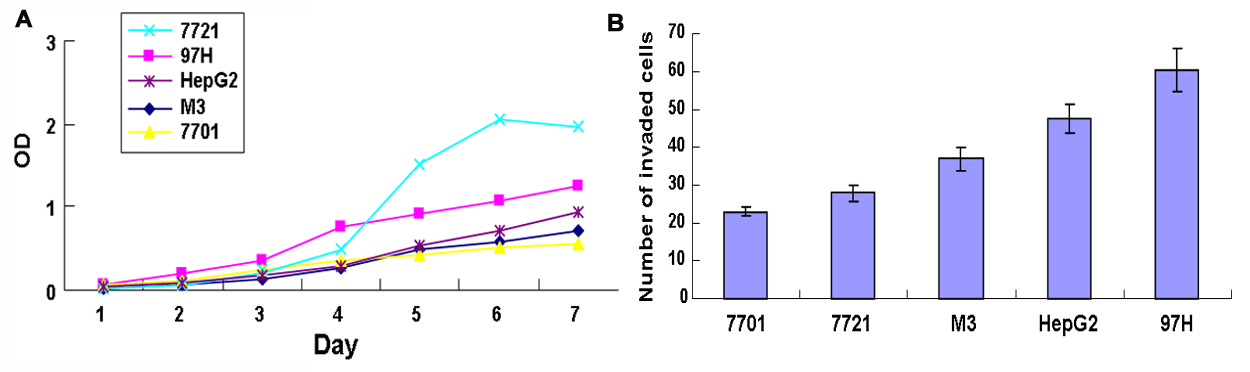

Supplement: Figure S1 — The proliferative and invasive abilities of five HCC cells in vitro. (A) Cell proliferation of five HCC cells was detected by MTT assay. (B) Cell invasion of five HCC cells was examined by Boyden invasion chamber. (TIF) [file pone.0065495.s001.tif]
