# Supplementary material for: FBX8 Acts as an Invasion and Metastasis Suppressor and Correlates with Poor Survival in Hepatocellular Carcinoma
Source: PLoS One. 2013 Jun 27;8(6):e65495. doi: 10.1371/journal.pone.0065495 (PMC3694991; doi:10.1371/journal.pone.0065495)
Supplement: File S1 — Table S1. Expression of FBX8 in HCC tissues, cirrhotic liver and adjacent normal liver tissues. Table S2. Clinical characteristics of 106 cases of HCC patients. Table S3. Univariate and multivariate analyses of individual parameters for correlations with overall Survival rate: Cox proportional hazards model. (DOCX) [file pone.0065495.s002.docx]

**Supporting Information**

Table S1 Expression of FBX8 in HCC tissues, cirrhotic liver and adjacent normal liver tissues

| Group | FBX8 expression | | | | Total |
| --- | --- | --- | --- | --- | --- |
|  | - | + | ++ | +++ |  |
| Noncancerous liver tissues | 5 | 30 | 49 | 36 | 106 |
| Cirrhotic liver  Adjacent normal liver | 0  0 | 1  6 | 10  21 | 23  45 | 34  72 |
| HCC tissues | 61 | 22 | 22 | 1 | 106 |
| HCC tissues with cirrhosis  HCC tissues without cirrhosis | 2833 | 1210 | 9  13 | 1  0 | 50  56 |

Noncancerous liver tissues vs HCC tissues Z= -8.684, P<0.001 (Wilcoxon Signed Ranks Test)

HCC tissues vs cirrhotic liver Z= - 5.047, P<0.001 (Wilcoxon Signed Ranks Test)

Adjacent normal liver vs HCC tissues Z= -7.038, P<0.001 (Wilcoxon Signed Ranks Test)

HCC tissues with cirrhosis vs HCC tissues without cirrhosis λ=1.986, P=0.575 (Chi-Square Tests)

Table S2 Clinical characteristics of 106 cases of HCC patients

| Characteristic | No. of Patients | % |
| --- | --- | --- |
| Age,y  <55  >=55 | 68  38 | 64.2  35.8 |
| Differentiation  Well  Moderate  Poor | 23  64  19 | 21.7  60.4  17.9 |
| Distant metastasis  Y  N | 69  37 | 65.1  34.9 |
| Dissemination  Y  N | 50  56 | 47.2  52.8 |
| Portalvein thrombosis  N  Y | 90  16 | 84.9  15.1 |
| Relapse  N  Y | 59  47 | 55.7  44.3 |
| Expression of FBXO8  Low  High | 81  25 | 76.4  23.6 |
| Cirrhosis  N  Y | 56  50 | 52.8  47.2 |
| Tumor size  <5cm  >=5cm | 50  56 | 47.2  52.8 |
| Gender  Male  Female | 92  14 | 86.8  13.2 |
| Serum AFP  <25ng/ml  >=25ng/ml | 37  69 | 34.9  65.1 |
| HBsAg  Negative  Positive | 21  85 | 19.8  80.2 |

Table S3 Univariate and multivariate analyses of individual parameters for correlations with overall Survival rate: Cox proportional hazards model.

| Variables | Univariate | | P value | Multivariate | | P value |
| --- | --- | --- | --- | --- | --- | --- |
|  | HR | CI (95%) |  | HR | CI (95%) |  |
| FBXO8 | 0.223 | 0.084-0.649 | 0.005 | 0.319 | 0.109-0.939 | 0.038 |
| Age | 1.022 | 0.566-1.847 | 0.942 |  |  |  |
| Gender | 2.459 | 0.764-7.915 | 0.132 |  |  |  |
| Portal vein thrombosis | 0.247 | 0.129-0.470 | <0.001 | 0.297 | 0.134-0.657 | 0.003 |
| Differentiation | 0.464 | 0.297-0.724 | 0.001 | 0.383 | 0.213-0.689 | 0.001 |
| Cirrhosis | 1.157 | 0.656-2.039 | 0.614 |  |  |  |
| Tumor size | 1.319 | 0.745-2.334 | 0.342 |  |  |  |
| Distant metastasis | 0.432 | 0.244-0.762 | 0.004 |  |  |  |
| Dissemination | 0.320 | 0.169-0.607 | <0.001 | 0.204 | 0.09-0.462 | <0.001 |
| Relapse | 2.065 | 1.162-3.671 | 0.014 |  |  |  |
| HBsAg status | 0.606 | 0.315-1.166 | 0.134 |  |  |  |
| Serum AFP | 0.824 | 0.462-1.471 | 0.513 |  |  |  |

Abbreviations: HR, Hazard radio; CI, Confidence interval.

|  |
| --- |
