# Supplementary material for: FBX8 Acts as an Invasion and Metastasis Suppressor and Correlates with Poor Survival in Hepatocellular Carcinoma
Source: PLoS One. 2013 Jun 27;8(6):e65495. doi: 10.1371/journal.pone.0065495 (PMC3694991; doi:10.1371/journal.pone.0065495)
Supplement: Methods S1 — Supporting material. (DOC) [file pone.0065495.s003.doc]

**Supporting Information**

**Material and Methods**

***HCC cell lines and clinical tissue specimens***

A hepatocyte cell line (HL-7702) and five HCC cell lines (HepG2, HCCL-M3, MHCC-97H, SMMC-7721 and QGy-7701) were purchased from the cell bank of Chinese Academy of Sciences. HL-7702 was maintained in RPMI 1640 (Hyclone, Logan city, USA) supplemented with 20% fetal bovine serum (FBS). Five HCC cell lines were maintained in RPMI 1640 (Hyclone, Logan city, USA) supplemented with 10% FBS. All the cell lines were in a 5% CO_2_ humidified atmosphere at 37℃. A total of 106 patients with primary HCC, who had undergone routine surgery at Nanfang Hospital affiliated with Southern Medical University from 2003 to 2005 year, were enrolled in this study. Clinical information of the samples in detail was seen in Table S2. Patients included 92 males and 14 females, of ages ranging from 27 to 76 years (mean, 50 years old). They were not pretreated with radiotherapy or chemotherapy prior to surgery. They were followed up for 5 years and their complete clinical data were collected. All primary HCC samples taken from the 106 patients were fixed in 10% formalin, embedded in paraffin, sectioned consecutively at 4 µm and stained by hematoxylin and eosin. Fresh HCC tissues and the corresponding normal tissues were collected from 20 patients who underwent CRC resection without prior radiotherapy and chemotherapy at the Department of General Surgery in Nanfang Hospital in 2009 year. These samples were collected immediately after resection, snap-frozen in liquid nitrogen, and then stored at -80℃ until needed. The histological types were assigned according to the criteria of the WHO classification system by three pathologists independently in a double-blinded manner.

***Evaluation of immunohistochemical staining***

The total FBX8 immunostaining score was calculated as the sum of the percent positivity of stained tumor cells and the staining intensity. The percent positivity was scored as ‘‘0,’’ 0%; ‘‘1,’’ 1–25%; ‘‘2,’’ 26–50%; ‘‘3,’’ 51–75% and ‘‘4,’’ >75%. The staining intensity was scored as ‘‘0’’ (no staining), ‘‘1’’ (weakly stained), ‘‘2’’ (moderately stained) and ‘‘3’’ (strongly stained). Both percent positivity of cells and staining intensity were decided in a double blinded manner. The staining of FBX8 was assessed as follows: (−) means a final staining score of <3; (+) a final staining score of 3; (+ +) a final staining score of 4; and (+ + +) a final staining score of ≥5, as shown in Table 1. This relatively simple, reproducible scoring method gives highly concordant results between independent evaluators[1,2]. Cutoff values for FBX8 were chosen based on a measure of heterogeneity by using log-rank statistical analysis with respect to overall survival. An optimal cutoff value was identified: Tumors with a final staining score 0~+ were classified as those with low expression of FBX8, and tumors with a final staining score ++~+++ were classified as those with high expression of FBX8.

***Real-time quantitative RT-PCR***

Total RNA was extracted using Trizol reagent (Invitrogen, Carlsbad, CA) and cDNA was synthesized by using an access reverse transcription system (Promega, Madison city, USA). Real-time PCR was performed using Mx3000P Real-time PCR System (Stratagene, La Jolla city), using the following thermal cycling profile: 95°C for 10 min, followed by 35 cycles of amplification (95°C for 10s, 60°C for 20s, 72°C for 34s). Relative quantity of target transcripts in each sample was expressed as N-fold differences relative to control, or 1×sample, and according to the equation: ΔΔCt=[Ct(target gene)-Ct(GAPDH)]experimental group-[Ct(target gene)-Ct(GAPDH)]control group. Here, the normal hepatic epithelial HL-7702 cells became “control group”. Human GAPDH gene amplified as an internal control. Each sample was tested three times and all other quantities were expressed as an n-fold difference relative to the corresponding control group. Primer sequences of FBX8 were as follows: forward 5’-GAT TCG CCA AAG GAA ATA GC-3’, reverse 5’-GTT ACA AGG TCA TCC AAG AC-3’.

***Western blot analysis***

Cells were washed twice with cold PBS and lysed on ice in RIPA buffer with protease inhibitors and quantified by BCA method. 50μg Protein lysates were resolved on 6% SDS polyacrylamide gel, electrotransferred to polyvinylidene fluoride membranes (ImmobilonP; Millipore, Bedford, MA) and blocked in 5% nonfat dry milk in Tris-buffered saline (pH 7.5). Membranes were immunoblotted overnight at 4°C with anti-FBX8 monoclonal antibody (Abcam), anti-actin antibody (Santa Cruz Biotechnology, Santa Cruz, CA), followed by their respective secondary antibodies. Signals were detected by enhanced chemiluminescence (Pierce, Rockford, IL).

***Plate colony formation array***

About 1× 10^2^ cells were added to each well of a 6-well culture plate. The cells were incubated at 37℃ for 14 d, then were washed twice with PBS and stained with Giemsa solution. The number of colonies containing 50 cells or more was counted under a microscope [plate clone formation efficiency = (number of colonies / number of cells inoculated) ×100%].

***In vitro motility assay***

Cells were cultured to 80% density and then digested by 0.25% of trypsin. 0.5×10^5^/ml of Cell suspension was made by RPMI-1640 without serum. Transwell chamber (Chemicon, USA) was used, in which microporous membranes with 8μm pore diameter were covered in the upper and lower chambers. The culture solution without serum was added into the upper chamber and incubated for 90mins. 200μl of cell suspension was then added into the upper chamber while 500μl of culture solution with 10% bovine serum was put into the lower one as the chemotactic factor. Cells were incubated for 24h at 37°C and 5％CO_2_. The numbers of trans-membrane cells were counted from five fields of vision under light microscope. Each experiment was repeated three times.

***Statistical analysis***

The expression of *FBX8* in HCC tissues compared to adjacent cirrhotic livers or normal livers was analyzed with Wilcoxon Signed Ranks Test. The correlation of *FBX8* expression to various clinicopathological parameters was evaluated with chi-square test. Survival analyses were performed according to the Kaplan-Meier method and compared by the log-rank test. MTT assay, plate colony formation assay, *in vitro* motility and invasion assays were tested using one-way ANOVA for factorial design. The SPSS 13.0 software was used for all statistical analyses and P<0.05 was considered significant.

1. [Chang, C.Y](http://www.ncbi.nlm.nih.gov/pubmed?term=Chang CY%5BAuthor%5D&cauthor=true&cauthor_uid=21996735). *et al.* (2012) Somatic LMCD1 mutations promoted cell migration and tumor metastasis in hepatocellular carcinoma. [*Oncogene*](http://www.ncbi.nlm.nih.gov/pubmed/21996735),**31**, 2640-52

2. Huang, J. et al. [Tiam1 is associated with hepatocellular carcinoma metastasis.](http://www.ncbi.nlm.nih.gov/pubmed/22573407) *Int. J. Cancer,* **132**, 90-100
